# Enabling novel paradigms: a biological questions-based approach to human chemical hazard and drug safety assessment

**DOI:** 10.1093/toxsci/kfad124

**Published:** 2023-12-22

**Authors:** Brian R Berridge, John R Bucher, Frank Sistare, James L Stevens, Grace A Chappell, Meredith Clemons, Samantha Snow, Jessica Wignall, Kelly A Shipkowski

**Affiliations:** Division of Translational Toxicology, National Institute of Environmental Health Sciences, Research Triangle Park, North Carolina 27709, USA; Retired (Division of Translational Toxicology, NIEHS), Hillsborough, North Carolina 27278, USA; Chapel Hill, North Carolina 27517, USA; Paradox Found Consulting Services, Apex, North Carolina 27523, USA; ICF, Reston, Virginia 20190, USA; ICF, Reston, Virginia 20190, USA; ICF, Reston, Virginia 20190, USA; ICF, Reston, Virginia 20190, USA; Division of Translational Toxicology, National Institute of Environmental Health Sciences, Research Triangle Park, North Carolina 27709, USA

**Keywords:** hazard assessment, predictive toxicology, safety evaluation, translational sciences, risk assessment

## Abstract

Throughput needs, costs of time and resources, and concerns about the use of animals in hazard and safety assessment studies are fueling a growing interest in adopting new approach methodologies for use in product development and risk assessment. However, current efforts to define “next-generation risk assessment” vary considerably across commercial and regulatory sectors, and an *a priori* definition of the biological scope of data needed to assess hazards is generally lacking. We propose that the absence of clearly defined questions that can be answered during hazard assessment is the primary barrier to the generation of a paradigm flexible enough to be used across varying product development and approval decision contexts. Herein, we propose a biological questions-based approach (BQBA) for hazard and safety assessment to facilitate fit-for-purpose method selection and more efficient evidence-based decision-making. The key pillars of this novel approach are bioavailability, bioactivity, adversity, and susceptibility. This BQBA is compared with current hazard approaches and is applied in scenarios of varying pathobiological understanding and/or regulatory testing requirements. To further define the paradigm and key questions that allow better prediction and characterization of human health hazard, a multidisciplinary collaboration among stakeholder groups should be initiated.

Many aspire to a future biomedical research endeavor that is much less dependent on animal testing than the one we have today. Prompting that aspiration are concerns about the human relevance of animal studies, their ethical justification, and their lack of scalability to support current higher throughput needs. Traditional approaches to hazard assessment for both chemicals and pharmaceuticals involve conducting standardized sets of animal studies collecting a broad range of biological endpoints—frequently including dozens of organ weight measures, histopathological tissue examinations, and clinical chemistry measures—to inform evidence-based decisions regarding the potential risk of human harm. These approaches are time-consuming, resource-intensive, and often expected by regulatory bodies across the globe. These regulatory bodies are often guided by legislation, such as the Frank R. Lautenberg Chemical Safety for the 21st Century Act (amendment to the EPA Toxic Substances Control Act) (U.S. Congress, 2016); Registration, Evaluation, Authorization, and Restriction of Chemicals by the European Commission (REACH) (European Parliament and the Council of the European Union, 2016), and the U.S. Federal Food, Drug, and Cosmetic Act to ensure the safe conduct of clinical trials. Much of this legislation is increasingly supportive of nonanimal approaches but efforts to characterize those approaches will likely be hindered without a better definition of the specific questions we are trying to answer (U.S. Congress, 2022). Examples of the growing interest in replacing animals in chemical and cosmetic testing include the European Commission’s response to a Citizen’s Initiative to accelerate phasing out animal testing (European Commission, 2023).

Provided the appropriate data, regulators are equipped to assess the likelihood of hazard or risk associated with chemical, biological, or physical agents (xenobiotics). Historically, however, data from traditional animal studies have been considered the best model for such assessments, and the applicability and appropriateness of other types of data have not yet been defined. The current animal-based hazard assessment approach is evidence-based, where the evidence and the questions asked are defined by their design and the endpoints collected. The biological questions inherent in general toxicity studies are broad and intended to cover the full spectrum of potential toxicological targets in a human-relevant biological modeling system. Studies intended to interrogate more specific forms of toxicity (eg, reproductive and developmental toxicity) are narrower in scope but, again, are defined by the endpoints measured.

There is considerable effort and investment across the globe in developing new approach methodologies (NAMs). NAMs are defined by EPA as any nonvertebrate animal technology, methodology, approach, or combination thereof that can be used to provide information on chemical hazard and risk assessment (EPA, 2023). They include *in silico*, *in chemico*, and *in vitro* assays and high-throughput and high-content screening methods (eg, genomics, proteomics, metabolomics) (Gwinn, 2020). Technical advances in NAMs that leverage our understanding of biology at the cellular and molecular levels, model biology in increasingly complex and *in vivo*-relevant ways and make use of human-derived cells provide support that our aspiration is achievable.

Alternatively, developing a nonanimal approach that can replicate the significant biological breadth of an animal study has been challenging, particularly because the specific biological questions being asked have not been articulated. Instead, animal studies routinely measure endpoints we believe are likely to provide insights into harmful effects. A usual repeat-dose animal hazard assessment study can include histological evaluation of 40+ tissues, terminal weights for 5–10 organs, and quantitative assessment of 10–20 serum chemistry and hematological parameters. A NAMs-based approach is not likely to replicate that breadth and complexity of biology; however, such replication may not be necessary.

Several recent biological frameworks, including “key characteristics” and “adverse outcome pathways (AOPs),” are intended to support the use of NAMs by leveraging our experiences characterizing toxicity in various organ systems in animal studies and human populations. Key characteristics represent common bioactivities of substances that cause cancer or are toxic to reproductive, cardiovascular, developmental, and/or other organ systems (Arzuaga *et al.*, 2019; Germolec *et al.*, 2022; Guyton *et al.*, 2018; La Merrill *et al.*, 2020; Lind *et al.*, 2021; Luderer *et al.*, 2019; Rusyn *et al.*, 2021; Smith *et al.*, 2016; *al.*, 2020). AOPs are a sequential chain of causally linked events that occur at different levels of biological organization, and that together lead to an adverse (ie, harmful) health effect (OECD, 2022). AOPs are “simplified representations of disease pathways” (Bal-Price and Meek, 2017), and specific assays can be developed and used to test the ability of a compound to elicit a response indicative of individual events. These frameworks provide a useful foundation for designing novel approaches but fall short of defining specific hazard assessment questions or a discrete biological scope for those questions. Although consensus on the definition and the appropriate scenarios for application of these 2 example frameworks has not yet been achieved, they do provide a biological scope for outcome-specific hazard assessments. However, the resulting biological scopes may prove constrained by existing evidence (eg, key characteristics) or be too dense for practical applications (eg, AOPs). Key characteristics are representative of “known knowns” where the harmful effects of most concern are the “unknown unknowns.” Alternatively, AOPs represent a mechanistic depth that would be difficult to fully represent in a collection of reductionist NAMs.

Hazard identification and characterization is distinct from, but fundamental to, a risk assessment that additionally considers who is exposed (population or individuals), and to what extent (level and duration). Definitions distinguishing these and other key terms are provided in Table 1. Several next-generation risk assessment (NGRA) frameworks have been proposed to support the integration of nontraditional biological and mechanistic data into risk assessments (Avila *et al.*, 2020; Ball *et al.*, 2022; Baltazar *et al.*, 2020; Dent *et al.*, 2021; Gilmour *et al.*, 2020; Middleton *et al.*, 2022; van der Ven *et al.*, 2020). However, these risk frameworks and efforts are often hypothesis-generating and do not generally define *a priori* the specific biological data needed to define hazard. NGRA frameworks would benefit from a better-defined set of fundamental biological questions that would inform more evidence-based decisions about risk. Defining these biological questions would provide a more focused biological scope for hazard assessment, enable flexibility in model/assay selection, and facilitate more predictive approaches to assessments of substances (see Figure 1). Additionally, such approaches provide an opportunity to use animal studies more purposefully.

**Figure 1.**
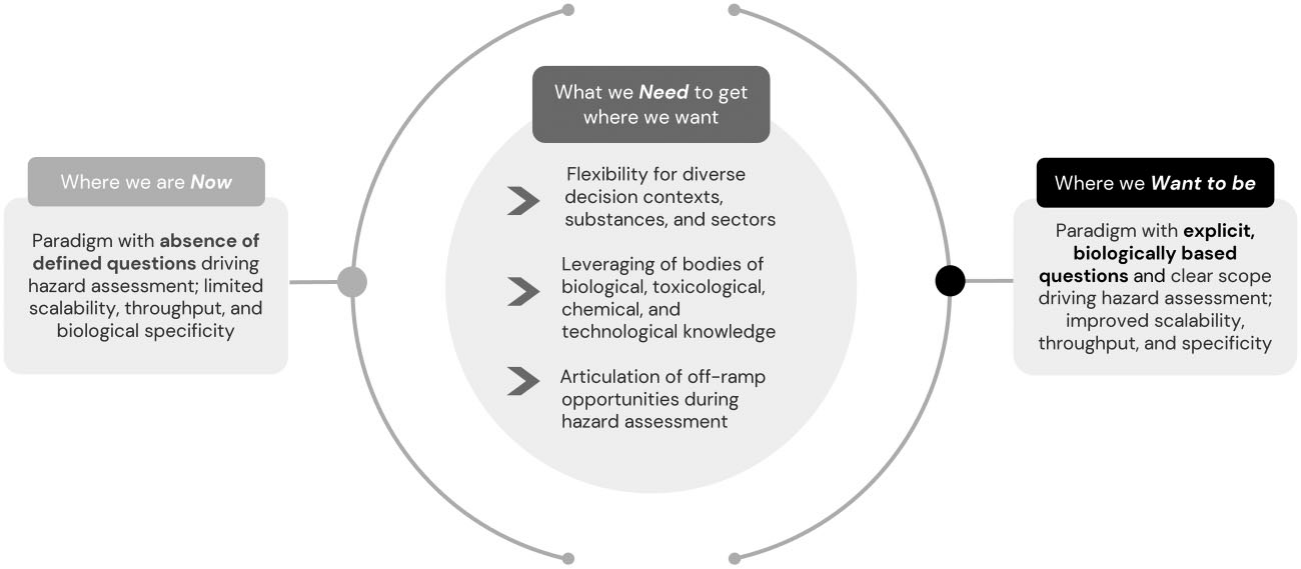
Current and potential future states of hazard assessment.

**Table 1.** List of key term definitions

Key term	Definition
Biological scope	The array of human biological systems and depth of biological understanding that must be considered during the assessment of a given substance to appropriately assess its hazard within a specific decision context.
Biological questions-based approach	A hazard assessment framework that is guided by a series of discrete questions related to the interaction of the substance of interest and a human-relevant biological system.
Decision context	The setting within which a hazard or safety decision is being made, which may include the sector (eg, agrochemical, personal care products), the stage of the substance life cycle (eg, product formulation, manufacturing), and/or regulatory requirements that might apply.
Hazard assessment	“A process designed to determine the possible adverse effects of an agent or situation to which an organism, system, or (sub)population could be exposed. The process includes hazard identification and hazard characterization. The process focuses on the hazard, in contrast to risk assessment, where exposure assessment is a distinct additional step.” (Barlow *et al.*, 2015)
Next generation risk assessment	“An exposure-led, hypothesis driven approach that has the potential to support animal-free safety decision making.” (Dent *et al.*, 2021)
Off-ramping	A process to discontinue further assessment activities when enough evidence has been amassed to gain sufficient biological understanding of a substance’s hazard (appropriate to the decision context).
Risk assessment	“A process intended to calculate or estimate the risk to a given target organism, system, or (sub)population, including the identification of attendant uncertainties, following exposure to a particular agent, taking into account the inherent characteristics of the agent of concern as well as the characteristics of the specific target system. The risk assessment process includes 4 steps: hazard identification, hazard characterization, exposure assessment, and risk characterization.” (Barlow *et al.*, 2015)
Safety assessment	For the purposes of this article, safety assessment refers to the hazard assessment of a drug or pharmaceutical that ultimately informs a patient risk assessment.

We propose that our fundamental understanding of a human host’s response to toxicity and our experience modeling those responses allow us to define a biological framework of hazard assessment questions that could be sufficiently flexible and scalable to apply across the decision-making continuum of product development. This biological questions-based approach (BQBA) could guide current efforts to define a novel hazard assessment paradigm that would more fully leverage both animal and nonanimal-based approaches, moving us more expeditiously toward meeting our aspiration.

## State of the science

We reviewed the current NGRA literature and NAMs-based approaches represented in 5 case studies for cosmetics, consumer products, environmental contaminants, occupational exposures, agricultural chemicals, and pharmaceuticals to inform the development of a BQBA, and a summary of selected references is presented here (Avila *et al.*, 2020; Ball *et al.*, 2022; Dent *et al.*, 2021; Middleton *et al.*, 2022; van der Ven *et al.*, 2020). Supplementary Table 1 presents common themes identified from this selection of literature.

NGRA frameworks (defined in Table 1) are designed to enable the use of more mechanistic and NAM-based data, with the required level of hazard characterization largely defined by the available data. Importantly, these frameworks do not prospectively define the specific biological hazard data needed for the risk assessment and instead rely upon data that are readily available, which undermines confidence in the completeness and outcome of the assessment. Generally, this has been less of a limitation with animal study-derived hazard data due to the confidence that the scope of biology present in a whole animal is a relevant surrogate for the scope of biology present in humans. Our proposed BQBA provides a framework that would allow better definition of the scope of biological data needed to conduct an NGRA, thereby increasing confidence in the outcomes. This framework would also guide the development of NAMs that would support an NGRA, as the specific information needed to conduct the assessment would be identified. Additionally, although Dent *et al.* (2021) focused on progressing toward an animal-free paradigm, the BQBA presented here is agnostic to modeling platform and could be useful in designing a novel animal-based approach. Our approach first defines the biological data necessary to determine a hazard, which would benefit any hazard modeling strategy.

Our experience in evaluating the toxicity of drugs and environmental chemicals can inform which biological systems are most likely to be affected. The depth of hazard characterization needed depends on the sector, the decision-making context, and any applicable regulatory guidance or requirements, providing flexibility in the application of different methods to generate relevant information. For safety testing of cosmetics, where animal testing is banned under European regulation, Dent *et al.* (2021) emphasized a focus on *in vitro* skin sensitization and the potential for systemic exposure, which is reasonably fit-for-purpose for cosmetics with topical administration. Alternatively, Avila *et al.* (2020) used a set of high-level questions currently addressed by pharmacology and toxicology studies (eg, safe first-in-human starting dose, maximum tolerated dose, consequences of chronic exposure) to highlight opportunities for NAMs to fill gaps in the existing pharmaceutical safety testing paradigm without replacing existing animal tests. Ball *et al.* (2022) outlined a tiered approach acknowledging that current NAMs applications are discrete and predominantly complements to animal studies. To completely replace animal studies, “…a large NAMs panel would be used simultaneously or consecutively…” The cosmetics NGRA proposed by Dent *et al.* (2021) is specific and biologically focused, providing a tractable means of designing a novel approach to address those fundamental questions. The higher-level questions proposed by Avila *et al.* have less biological definition and are more challenging to address with NAMs. The “large NAMs panel” mentioned by Ball *et al.* has yet to be defined, which is the primary limitation in a broader and more impactful use of NAMs.

Thus, despite general similarities in tiered approaches, differences in the scope of the biological considerations relevant to hazard characterization are driven by data needs that are specific to each overall context. As noted above, when relevant data provide a sufficient answer to a predefined biological question, the assessment can conclude or move forward to answer additional biological questions. These off ramps were represented in case studies by van der Ven *et al.* (2020) and Dent *et al.* (2021), in which both noted that low exposure levels of certain chemicals eliminated the need for further consideration of hazard.

## Biological questions-based approach

**Figure 2.**
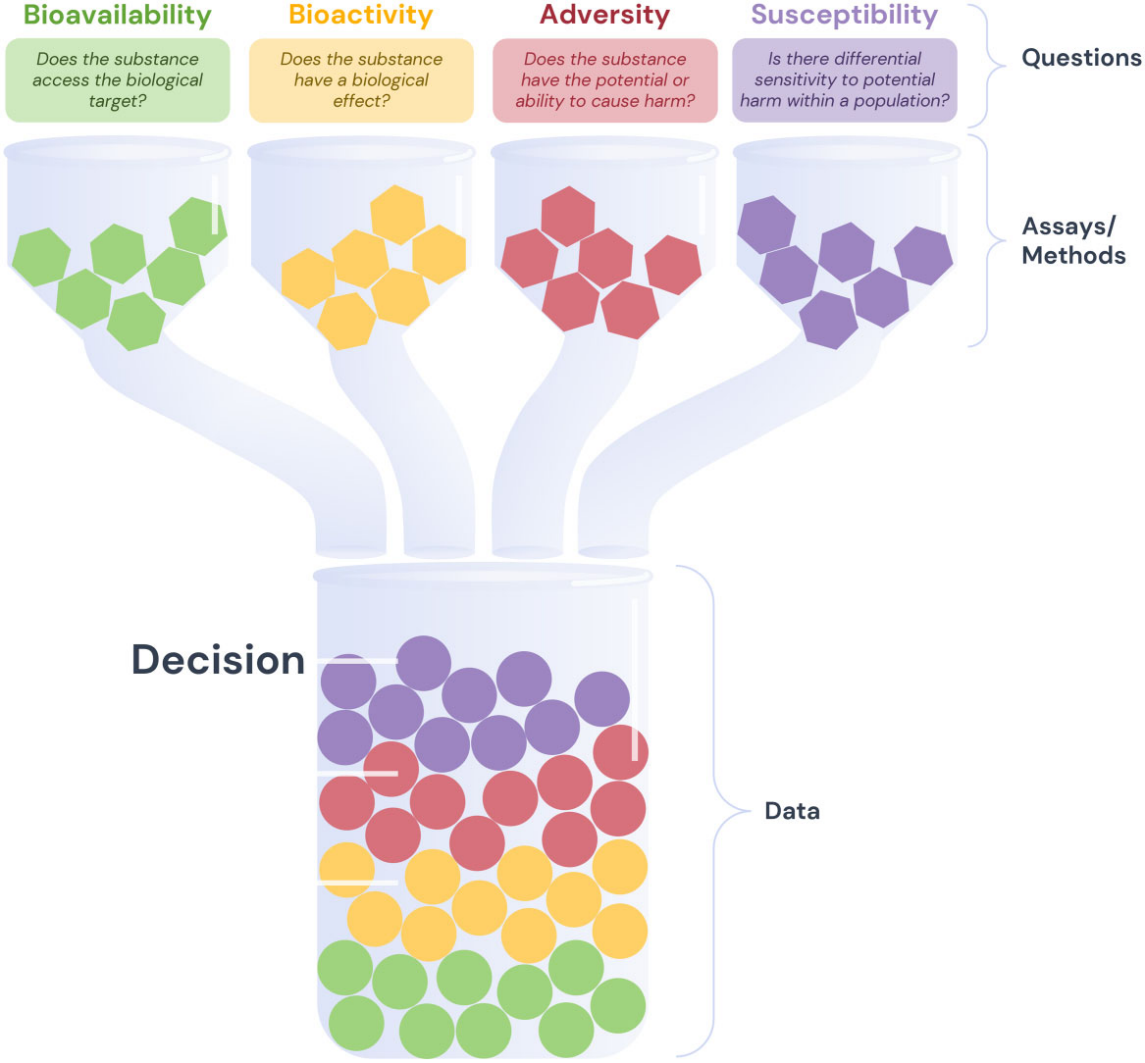
Graphical depiction of a BQBA. Questions articulated across the 4 biological pillars drive selection of appropriate assays, models, or methods to generate data that will inform a hazard decision. Assays and methods to assess each pillar are represented by hexagons. The outputs from those assays are represented by circles in the large beaker. The collection of assay data informs decisions related to the potential hazard of the substance.

The above review of trends emerging from current risk assessment frameworks revealed areas of consensus as well as differences in the depth and scope of biological understanding necessary to inform human health protective hazard decisions. Several fundamental biological considerations emerged from our review. Those key biological considerations, or “pillars of hazard,” were bioavailability, bioactivity, adversity, and susceptibility, providing a framework around which to define specific questions that would be addressed by a hazard assessment. To be harmful, a substance must be “bioavailable” such that it has the opportunity to encounter tissue(s) and/or cells in the host. The substance must also induce a host response or be “bioactive.” The responses of primary interest are those that lead to an adverse outcome (ie, a hazard). Finally, variation in response following exposure to a substance influenced by factors such as life stage, sex, socioeconomic status, an existing morbidity, or genetics (ie, susceptibility) represents a critical context for the previous 3 concepts. Clearly defined questions for each pillar would enable decision-makers to define the most appropriate data required for their purposes. For each of these pillars, some scope of data is required to conduct an assessment. The 4 pillars are interdependent, but their application in an assessment should be unique to the substance of interest. The proposed framework is presented in Table 2 and depicted in Figure 2.

**Table 2.** Key questions and example secondary questions by pillar

Pillar	Key/primary question	Example secondary questions	Example assessment/tests/strategies
Bioavailability	Does the substance access biological systems?	Is this substance bio-persistent or bio-accumulative? Does the substance produce a persistent metabolite? How much of the substance is bioavailable in various tissues, including locally to epithelial surfaces? Does the substance enter circulation? Can the substance enter target cells/tissues (eg, cross the blood-brain barrier)?	Physicochemical properties (eg, molecular weight, solubility, pKa, logP) ADME
Bioactivity	Does the substance induce a host response?	Is the effect measurable? In what tissues/cells does the bioactivity occur? At what dose does the bioactivity occur? Is the host response specific or nonspecific? Are there consequences of this response in other organ systems?	Receptor screens QSAR and/or read-across approaches Cytotoxicity Key characteristics-based assessments
Adversity	Does the substances have the potential to cause harm?	How does the bioactivity relate to known mechanisms or modes of action of toxicity? Is the effect reversible? Is there an adaptive response after the first dose/exposure? Are defined key characteristics of toxicity observed?	Functional agonist or antagonist activity Cell/tissue health Organ system health AOP-based assessments
Susceptibility	Is there differential sensitivity to potential harm within a population?	Is there an increased risk for adversity relative to the general population that is due to intrinsic factors such as Particular life stages (ie, developing fetus, puberty, etc.)? Certain genotypes/genetic polymorphisms?	DART and DNT testing Genetically diverse cell-based testing systems Integration with other relevant hazard information

Additional information about each pillar and its associated questions is outlined in the following section.

## BQBA pillars

### Bioavailability

The ability of a substance to gain entry and interact with a biological system when external exposure has occurred—which is presumed in this framework—is fundamental to its potential to cause harm. In animal studies, direct measure of the concentration of a substance or its metabolite over time in plasma, in specific organs, or in excretory substrates are used to assess bioavailability. Nonanimal approaches used in hazard assessment must address and represent the complex biology of *in vivo* ADME. Substances that lack local or systemic bioavailability can be assumed to pose little to no hazard to health. Although bioavailability does not inherently represent hazardous activity, it may facilitate harm to the full spectrum of tissues and organs in a mammalian system. An exception to a dependence on systemic bioavailability to induce harm are substances that elicit a biological response through a mechanical interaction with an epithelial surface such as nanoparticles in the respiratory system or abrasive irritants on the skin.

The bioavailability of a novel compound can be evaluated in many ways, beginning with characterizing the physicochemical properties of the substance (eg, molecular weight, lipophilicity, reactivity) that will significantly influence the likelihood and magnitude of its bioavailability. The biological fate of a substance is determined by its absorption, distribution, metabolism, and excretion (ie, ADME properties) in a living system. Factors that limit systemic bioavailability include poor absorption and rapid metabolism and excretion. Bioavailability can be computationally modeled using physicochemical properties like molecular weight, pKa, log P, etc. A variety of *in vitro* assays modeling key biological processes like intestinal absorption, protein binding, and intrinsic hepatic clearance can be used to predict the likelihood of systemic bioavailability, or it can be directly tested in animals (Chiba *et al.*, 2009; Volpe, 2020).

Different approaches (eg, computational, *in vitro*, *in vivo*) offer different types of data and throughput capability when answering the question, “Is this compound bioavailable?” For example, depending on the proposed uses and potential for human exposure, physicochemical properties of a substance may provide sufficient information to predict systemic bioavailability or suggest that an assessment of bioaccumulation/tissue burden is needed. All 5 of the case studies we reviewed provided some assessment of systemic bioavailability by pharmacokinetic modeling or by considering ADME data and physicochemical properties (Supplementary Table 1). Although the term bioavailability was not universally applied, the consistency of bioavailability concepts across contexts reflects the importance of this pillar in understanding both systemic and local tissue hazard.

### Bioactivity

Bioactivity represents the potential for a substance to elicit a response in cells, tissues, or organs. Bioactive substances generally induce a response from the host and may directly interact with DNA or proteins or initiate a molecular signaling response by binding to a receptor or altering an enzyme mediator. However, bioactivity is not always detrimental or harmful.

Traditional animal studies provide data on bioactivity through measures of alterations at the molecular up to the organ level. For example, binding of a chemical to macromolecules, such as DNA or proteins, can be measured in biological matrices from animal studies. Such molecular interactions can result in alterations in gene and protein expression, which can also be measured in samples from animals. Such molecular responses to a chemical can result in changes to cellular signaling, and subsequent tissue-level effects such as changes to organ weights and function and histopathological changes. A non-animal or NAM-based approach would need to define the amount of typical *in vivo* biology necessary to reliably detect and characterize host bioactivity.

Like bioavailability, bioactivity can also be predicted from previous experiences using in silico methods, such as computational modeling based on structural properties of a substance (eg, quantitative structure-activity relationship [QSAR] modeling). Chemical read-across methods can also be applied to predict bioactivity, based on similarities in physicochemical properties (Patlewicz and Shah, 2023). Bioactivity can also be directly evaluated in a diverse array of cell-free, *in vitro,* and *ex vivo* models, including the molecular-level changes described above. The selection of assays may be guided by *in silico* QSAR predictions and the context of use; for example, there are assays and models designed to predict or measure the ability of a substance to perturb nuclear receptors that modulate the endocrine system.

A significant challenge to bioactivity assessment is defining the appropriate biological scope and level of complexity of the test system needed for the intended context of use. Animal studies represent the full breadth and complexity we expect in a human biological system but lack the scalability of non-animal approaches. An example of a more reductionist bioactivity assessment approach is the predefined panels of known pharmacological targets used by pharmaceutical companies to screen unintended “secondary” pharmacological bioactivity and the potential for off-target liabilities (Bowes *et al.*, 2012) often prior to animal studies. Other approaches evaluate “cell health” in immortalized or primary cells of varying types. More complex modeling systems, such as 3-dimensional organotypic models, enable the assessment of tissue and organ level biology.

There are a growing number of bioactivity modeling systems available but little definition of the scope of bioactivity assessments necessary to conduct a reliable assessment. Also, recognizing that the endpoints measured in more reductionist bioactivity assays are often different than those we measure in animal studies, it would be useful to define which endpoints are necessary to inform definition of hazard. These are fundamental gaps that will need to be defined to support a broader application of NAMs-based hazard and risk assessment. Bioactivity was examined in all 5 case studies, as represented by the yellow text in Supplementary Table 1. Again, the consistent consideration of this pillar across sectors underlines its importance in assessing hazard.

### Adversity

A biological effect may be adverse or non-adverse. “Adverse” implies that a substance induces a harmful effect, generally defined as any change that impairs performance (ie, functional capacity of an organ or system) or the capacity to compensate for stress and/or repair damage, or that renders a biological system or whole organism susceptible to other stressors (Engelhardt and Dorato, 2021). Such characteristics are expected to have a detrimental effect on growth, development, and/or lifespan. Adverse biological effects include loss of cells, perturbation in function of a major organ, or abnormal proliferation (eg, carcinogenicity). Key to establishing adversity is distinguishing an adverse response that causes harm from an adaptive response that initiates a change in biology that is not harmful to the host. That distinction may be dose- or duration of exposure-dependent. As such, understanding adversity requires a greater degree of characterization than the first 2 pillars.

Most host reactions to a xenobiotic occur on a continuum of adaptive responses to overt pathology with loss of cell, tissue, or organ function. Adversity has traditionally been informed by results from animal testing, where it may be recognized as pathologic changes in morphology or organ dysfunction. Relative to bioactivity or bioavailability, the evaluation of adversity brings unique challenges to defining a tractable BQBA because of the potential breadth or complexity of biological representation required in the test system. Bioactivity of a substance must be assessed in the appropriate system and context (eg, a target tissue of concern with human-relevant biology) to understand if it is likely to be adverse. Furthermore, a greater understanding of the target tissue’s pathobiology is needed to define appropriate endpoints. In traditional (ie, animal-based) test systems, apical adverse outcomes are usually characterized (ie, the pathologic end of the continuum). More proximate events are often more accessible in a cell or tissue-based modeling system—a key strength—but an understanding of the relationship between mechanistic events and an adverse outcome is necessary for such mechanistic events to be used for prediction. For example, a causal relationship between a cellular or molecular event and an adverse outcome must be demonstrated, replicated, and accepted for such an “upstream” event to be considered indicative of a strong potential for the adverse outcome to occur.

Designing models and assays that address defined key characteristics of toxicities and/or molecular events within an AOP could guide a defined set of tests to understand the likelihood of a substance to cause specific types of adversities. A simple example of this is testing compounds in metabolically competent assays for genotoxicity, a known key event in carcinogenesis. These assays can be selected based on their appropriateness for the context of use (eg, evaluating key events that are related to developmental effects and endocrine disruption are important in pesticide testing) or on results from bioavailability and bioactivity evaluations (eg, a substance that has bioactivity on cardiac contractility-relevant Β_1_ adrenergic receptors could be further tested for higher order events such as alterations to cardiomyocyte or whole organ function).

Understanding and defining the relationship between a key biological event and an adverse outcome is essential. For example, a substance that is bioactive in certain key characteristics or a single key event in an AOP may not present significant probability for the adverse outcome of concern, while bioactivity in other key characteristics or key events could reliably lead to the adverse outcome. Assessment of adversity is a potential source of considerable uncertainty unless we can better define thresholds between adaptive and maladaptive responses. Adversity was universally considered in the 5 case studies (Supplementary Table 1, red text), underscoring the importance of understanding adversity to determine hazard.

### Susceptibility

Adversity may be host dependent. Accordingly, an important consideration in hazard assessment is characterization of the variability in response to exposure within and across different populations and life stages. Assessments should account for sensitive individuals or populations in addition to “typical” individuals (ie, healthy adults). Susceptibility can modify the adversity of a substance and, thus, its hazard.

Critical developmental stages represent sources of increased susceptibility, as do genetic variations that modify xenobiotic metabolism or lead to immunologic hypersensitivity. Currently, early life development and sex are most commonly assessed as sources of susceptibility, although there are other sources such as genetic predisposition, advanced age, and pre-existing disease or co-morbidities. Traditional animal studies address susceptibility primarily by studying specific developmental stages, or by creating genetic models of human disease or traits. Additionally, limited understanding of susceptibility is typically addressed by the inclusion of standard uncertainty factors in risk assessments using animal data. A BQBA that includes questions about specific susceptibilities as they relate to the population and/or outcome of interest would ensure broader protection for human populations, including those likely to be most vulnerable. For example, in cases in which adversity is related to development, sensitive windows of development could be evaluated in models that represent early developmental stages of organ development. Discussions of susceptibility are also addressed in the case studies summarized in Supplementary Table 1 (purple text); however, only 2 of the 5 case studies explicitly discuss considerations of susceptibility, with one outlining NAMs to assess genetic and immune diversity and the other asserting the need to consider population variability that impacts individual susceptibility when determining hazard.

### Key questions

In our proposed paradigm, each biological pillar could be represented by a primary question that, depending on the answer, is complemented by secondary questions that provide additional information about the pillar for the hazard assessment. Flexibility in the questions and how they are answered is fundamental to the general usefulness of this approach. The key point is that there are specific questions articulated rather than a collection of assays without a defined framework. A BQBA enables decision-makers to articulate and answer questions appropriately for their unique context. Secondary questions will vary by substance, sector, and decision-making context, although some examples are presented.

It is not the intent of this article to define all the relevant hazard assessment questions, but to propose that a biological framework exists (eg, the pillars), that questions could be defined by relevant experts, and that addressing these questions would significantly support efforts to develop novel approaches to hazard assessment with broader applicability to contemporary challenges.

## Potential implementation of a BQBA

### BQBA in a continuum of sectors and decision-making contexts

The diversity of sectors and contexts across which human hazard assessments are conducted poses a challenge in developing flexible paradigms that apply across settings. A BQBA could be agnostic to sector. A multidimensional continuum of evidence gathering and decision-making occurs throughout a substance’s life cycle. The data supporting hazard decisions can be framed in a triaxial manner: one axis defined by the stage of the substance life cycle in which hazard is considered; another axis defined by the biological understanding appropriate to characterize the hazard, including types, volume, and complexity of data; and the third axis representing the level of tolerance for uncertainty of the data as it relates to predictability of human health effects. Decision-makers in specific sectors may leverage this continuum in their unique decision-making process. Each stage of the substance life cycle takes place at a different point on that continuum and, therefore, requires a different level of evidence to sufficiently characterize hazard and protect human health.

Accordingly, the specific decision-making context defines the level of evidence needed to gain sufficient biological understanding of a substance’s hazard, which represents a practical “off-ramping” or triaging opportunity for decision-makers. The data requirements to demonstrate a lack of hazard for a pesticide, or to move a novel pharmaceutical into clinical trials, are very different from those needed to screen thousands of compounds to identify those with the highest potential for a favorable risk: benefit ratio versus those most likely to cause harm. Although much of the focus on getting support for novel approaches to human hazard assessment is on regulators, there are many decisions related to safety made by various stakeholders along the development life cycle of a product that are “pre-regulatory” and not well supported by the usual animal study-based approaches. Current approaches to hazard assessment are not amenable to use in early stages of chemical and pharmaceutical product development or to screen large numbers of untested substances in our environment. A useful biological framework would provide the flexibility to gather a fit-for-purpose level of hazard information and more than 1 way to generate that information.

### BQBA case studies

To understand how a BQBA may be defined and applied in hazard assessment, 3 examples of toxicities with varying requirements regarding safety testing and hazard identification were considered. These “exemplars” are briefly described here and are further detailed in the supplementary materials (Supplementary Tables 2–4).

Cardiovascular toxicity is a key contributor to drug safety-related development attrition. Our understanding of the biology of the cardiovascular system and how it responds to injury enables us to define a set of relevant questions and to design modeling systems to answer those questions (see Supplementary Table 2). “Impairs cardiac contractility” is a key characteristic of a cardiovascular toxicant and an AOP has been defined that links blockade of L-type calcium channels on the cardiomyocyte cell membrane to contractile heart failure. Accordingly, a reasonable biological hazard assessment question is: “Could exposure to substance X result in changes in cardiac contractile function in humans?” Answering that question would require defining the relevant biological scope of an appropriate modeling system(s), recognizing that there are many causes of contractile dysfunction in addition to a blocked L-type calcium channel (eg, cardiomyocyte necrosis, myocardial fibrosis, mitochondrial dysfunction, disruption of the myofibrillar contractile apparatus). Our mechanistic understanding of cardiac contractility and the rapidly expanding ways of modeling key elements of that biology represent an opportunity to define fit-for-purpose assessments. The most “human-relevant” assessment might be based on an animal model, although at the expense of considerable time, expense, and throughput. Models of more salient biological features of cardiac contractility might be more scalable and fit for purpose. Similar approaches could be taken across other important organ systems.

We also considered the 4 pillars in the context of an outcome for which specific regulatory guidance exists. Assays preselected by EPA for testing the endocrine disruption of pesticides (EPA, 2009, 2011, 2015; Stoker and Kavlock, 2010) were mapped to the 4 pillars presented herein to understand how the current testing strategy fits with the BQBA (see Supplementary Table 3). This second exemplar demonstrates an alignment with the BQBA (the question being, “Does substance X disrupt the endocrine system?”), while also showing how the testing battery could be refined with a more defined biological scope (ie, by adding a bioavailability information requirement).

The 2 examples above represent well-characterized biological spaces. For the third exemplar, we considered neurobehavioral/neurotoxic outcomes from developmental exposures, a hazard currently evaluated with insensitive and technically challenging animal models (Behl *et al*., 2019, see Supplementary Table 4). Alternative assay batteries have been proposed and continue to be tested, refined, and validated. Gaps in the understanding of the pathobiology in this case may present challenges in addressing all pillars in the BQBA (discussed further in the Limitations section). However, framing the assay development or testing requirements around specific questions (eg, “Does this substance cross the blood-brain barrier?” “Does this substance bind to a specific neurotransmitter that is related to a known neurobehavioral effect?”) will benefit the future of testing for such outcomes in product safety research and development as well as in regulatory development.

In all 3 examples, a BQBA provides a structured framework to guide the selection of fit-for-purpose assays that directly address data needs. We have proposed specific assays or assay types for the 3 examples described above according to the 4 pillars to demonstrate the potential implementation of BQBA across the 3 different scenarios.

## Limitations

The articulation of specific biological questions to guide hazard assessment, as discussed above, requires knowledge of the underlying biological systems appropriate to the decision-making context. It is unlikely that the breadth and complexity of traditional animal-based testing can be fully replicated, however, there is an opportunity to strategically replicate the key components of those models in a more human-relevant way. Where biological systems are less defined and mechanisms underlying adverse outcomes are unclear, the application of the BQBA will be limited, although the pillars can provide a structure and framework for gathering necessary information. In areas where toxicological mechanisms or modes of action are less understood, decision-makers and hazard assessors may be limited in their ability to articulate specific questions, and the use of traditional, observation-based animal studies may continue to be most appropriate. As the breadth and depth of biological understanding continues to progress, we anticipate that the BQBA can be more progressively applied.

## Call to action

Hazard assessors have many motivations for developing a novel paradigm that is more efficient and accommodating in a broader range of decision contexts. We contend that many of the enabling concepts, principles, and capabilities exist. We know where we are trying to make hazard decisions, and many stakeholder groups are willing to adopt a different model. Models of NGRA provide a useful and relevant risk assessment framework within which a “next-generation hazard assessment paradigm” might fit. We have a growing portfolio of modeling capabilities and could recruit more by defining gaps in our current portfolio. We propose that our primary obstacle to fully leveraging these resources is a failure to define the questions we are trying to answer and the biological context for those questions.

The observational nature of our usual animal study-based approaches to hazard assessment has allowed us to avoid articulating the specific questions we are asking in those studies, and there are many of them. The current surrogate for specific questions is a list of tissues we routinely weigh and/or examine histologically and a set of biochemical and hematologic endpoints we measure (ie, the endpoints define the questions rather than the questions defining the endpoints). Accordingly, we struggle to move away from our traditional approaches because we cannot design an assay to answer a question that we have not articulated.

Defining and adopting a novel assessment paradigm requires that we develop a discrete set of human health-relevant questions, define a finite number of organ system targets for those questions, identify the ways toxicity manifests within those targets, and define the endpoints we would measure to represent those manifestations. This represents a type of problem formulation to precede laboratory testing or computational modeling activities. The goal is to define the biological scope and substrate for addressing the problem. The biological scope can be defined along the continuum of biological complexity from molecular targets, to cells, to tissues, and to organs and organ systems, enabling a spectrum of fit for purpose, adaptable modeling capabilities that can be applied in various decision contexts. The scope of questions we articulate should leverage our experience in characterizing hazards (eg, “In which target organs and cells is exposure or dose-limiting toxicity most likely to occur?”), our understanding of toxicologic modes of action (eg, “What are the usual pathways of injury to target cells?”), and our knowledge of how injury is manifested in the cells, tissues, and organs of most interest.

Defining the relevant questions and their respective biological scope will require a multidisciplinary collaboration among the various stakeholder groups interested in hazard and safety assessment. We will need to use our experience to agree on the most relevant target organs/tissues/cells. We will need pathobiologists to define the spectrum of ways that those targets respond to toxic injury. We will need pathobiologists, toxicologists, and cell biologists to define relevant endpoints. We will need risk assessors to help define an interpretive scheme to support the decisions made for those endpoints. It is likely we will also need assay developers to design modeling systems to fill gaps in our current portfolio.

We believe that, collectively, we have the relevant knowledge, experience, and capabilities to define these questions and the biology we will need to model to answer them. It is time to bring this multidisciplinary community together and to take a more biologically based approach to define this new paradigm in which we will have the confidence to make more evidence-based decisions along the decision continuum.

## Supplementary Material

kfad124_Supplementary_Data
