# Supplementary material for: Enabling novel paradigms: a biological questions-based approach to human chemical hazard and drug safety assessment
Source: Toxicol Sci. 2023 Dec 22;198(1):4–13. doi: 10.1093/toxsci/kfad124 (PMC10901149; doi:10.1093/toxsci/kfad124)
Supplement: kfad124_Supplementary_Data [file kfad124_supplementary_data.docx]

**Enabling novel paradigms: A biological questions-based approach to human chemical hazard and drug safety assessment**

**Berridge, B.R.**^a^***, J. Bucher**^b^**, F. Sistare**^c^**, J. Stevens**^d^**, G. Chappell**^e^**, M. Clemons**^e^**, S. Snow**^e^**, J. Wignall**^e^**, K.A. Shipkowski**^a^

^a^Division of Translational Toxicology, National Institute of Environmental Health Sciences, Research Triangle Park, North Carolina 27709, USA

^b^Retired (NIEHS), Hillsborough, North Carolina 27278, USA

^c^Chapel Hill, North Carolina 27517, USA

^d^Paradox Found Consulting Services, Apex, North Carolina 27523, USA

^e^ICF, Reston, Virginia 20190, USA

***Corresponding Author:**

Brian R. Berridge

[brberridge@b2pathologysolutions.com](mailto:brberridge@b2pathologysolutions.com)

**ORCIDs**

Brian Berridge – 0000-0003-1988-2819

John Bucher – 0000-0002-6795-8518

Frank Sistare – 0000-0001-7218-3756

James Stevens – 0009-0006-9593-5299

Grace Chappell – 0000-0002-7543-8336

Meredith Clemons – 0000-0002-7048-9281

Samantha Snow – 0000-0003-1812-8582

Jessica Wignall – 0000-0002-2947-7879

Kelly Shipkowski - 0000-0002-1781-4583

# Supplementary Materials

Table S-1. Case Studies: Recently Published Approaches for NGRA and Their Relevance to the BQBA

| **Reference and Context of Use** | **Approach** | **Notes**^a^ |
| --- | --- | --- |
| Avila et al. (2020)  Drugs/ pharmaceuticals | *Traditional*  Discusses integration of NAMs assays into the existing FDA regulatory framework and guidance. | - Bioavailability: Applies PK models to assess biological interactions. - Bioactivity: Discusses NAMs that could be leveraged to identify likely biological targets. - Adversity: Outlines critical organ systems for identifying on- and off-target adverse effects. - Susceptibility: Provides NAMs to identify rare effects influenced by genetic or immune diversity. - Biological Scope: Notes the organ systems for consideration in risk assessment. No discussion of selecting those most relevant to the context. |
| Ball et al. (2022)  Industrial/ consumer chemicals | *Questions-based*  Provides a 3-tier framework for incorporating NAMs under REACH for regulatory hazard decisions by assessing the acceptable hazard profile for a given situation. | - Bioavailability: Considers ADME in Tiers 1 and 2 of the proposed framework. - Bioactivity: Assesses bioactivity in Tier 2 of the proposed framework. - Adversity: Assesses adverse effects in specific organs and organ systems in Tiers 2 and 3 of the proposed framework. - Biological Scope: Notes that all tiers of the framework may not be necessary to reach an acceptable hazard profile, although “acceptable” is not defined in biological terms. |
| Dent et al. (2021)  Cosmetic ingredients | *Questions-based*  Argues for a hypothesis-driven NGRA approach that is anchored in International Cooperation on Cosmetics Regulation (ICCR) principles and predicts adverse effects from cosmetic ingredients. | - Bioavailability: Discusses physicochemical data, in silico PBK models, and read-across data to assess internal exposure and metabolites. - Bioactivity: Uses in chemico, in silico, and in vitro data with predicted internal exposure to derive a bioactivity exposure ratio (BER). - Adversity: Assumes large BERs indicate little likelihood of adverse events. Notes the limitations of in vitro and in silico tools for distinguishing adverse and adaptive responses. - Biological Scope: Emphasizes “adequate precision” but does not explain how to define that by context. |
| Middleton et al. (2022)  Consumer products | *Traditional*  Provides a NAM toolbox to assess hazard of consumer goods. Proposes a NAM substitution for the typical animal assay approach. | - Bioavailability: Uses a PBK model to estimate the concentration of a substance in the body. - Bioactivity: Applies three in vitro platforms, in tandem with the PBK, to derive a BER. - Adversity: Uses BER distributions to assess inherent risk level (low or high) of a chemical. - Susceptibility: Notes that future model iterations need to integrate population variability in hazard consideration. - Biological Scope: Provides no discussion of how to scope the amount of biological information necessary to make a determination. |
| van der Ven et al. (2020)  Fungicides | *Questions-based*  Provides an approach for assessing hazard identification using existing NAMs tools. Advocates for mechanistic-driven method selection rather than traditionally preselected endpoints. | - Bioavailability: Applies the Lipinski rule of five to assess bioavailability. - Bioactivity: Applies in silico tools to assess bioactivity and integrates results from in vitro studies to confirm results from bioactivity assays. - Adversity: Notes the need for further assessment of adversity. - Biological Scope: Emphasizes use of appropriate mechanistic evidence across a range of organ systems, but information on how to select appropriate systems is not provided. |
| FDA = Food and Drug Administration; NAMs = new approach methodologies; NGRA = next-generation risk assessment; PBK model = physiologically based kinetic model.  ^a^Text color denotes the following topics based on the biological questions-based approach pillars: Green text indicates information related to bioavailability; yellow text indicates information related to bioactivity; red text indicates information related to adversity; purple text indicates information related to variability and susceptibility. | | |

**Expansion of Potential Implementation of BQBA: Three Exemplars**

## Exemplar 1: Application of a BQBA to evaluate potential cardiovascular toxicity by candidate pharmaceuticals

The heart is a primary target organ of concern in safety assessment, with a finite number of ways that the heart can “fail” including loss of rhythmicity, loss of ability to generate contractile force, loss of ability to respond to changes in workload, failure to maintain unidirectional flow of blood, etc. Each of these “failure modes” can be a focus for questions in a safety assessment. To define a relevant test system for any particular failure mode, understanding the scope of the biology involved is necessary.

Cardiac contractile failure or dysfunction is a useful exemplar. Cardiac contractility is fundamental to the function of the heart specifically and the cardiovascular system generally. It is designed to respond to changes in demand or workload and represents a basic physiological function of the heart. A change in basal contractile function beyond a normal dynamic range or an inability of the heart to respond to changes in workload are pathologic. Changes in cardiac contractile function can result from direct or indirect causes—including myocardial injury with tissue remodeling (e.g., fibrosis) and changes in hemodynamic preload (ventricular blood volume) or afterload (peripheral resistance), autonomic signaling, catecholamine concentrations, cardiomyocyte calcium handling, and/or mitochondrial ATP production—all of which represent potential modes of action for cardiac contractile toxicants.

A BQBA for cardiac contractility would not only include fundamental questions about bioavailability but would also consider contractility-related bioactivities at the cellular level and, potentially, effects at the tissue and organ level depending on the decision-making context. For example, a calcium transient assay might be sufficient for a screening but a measure of whole heart contractile function might be necessary for regulatory approval. Questions would include those related to all the potential modes of action mentioned above (i.e., calcium handling, ATP production, myocardial injury) which could be supported by a portfolio of test systems that would evaluate a substance’s likelihood of disrupting these processes. A complete evaluation of cardiovascular safety or hazard would include assessments that address discrete questions for all the potential failure modes. The questions defined by a BQBA for any particular toxicological target organ of interest could be applied using a tiered decision tree. An example decision tree for contractile cardiotoxicity using a BQBA could look like this:

- Is this substance bioavailable?
  - If not, the answer to the primary question is “no” and further assessment of cardiac endpoints is not necessary (an “off ramp”).
- If this substance is bioavailable, is it active on biological targets that mediate cardiac contractile function?
  - If not, the answer to the primary question is “no” and further assessment of cardiac endpoints is not necessary (an “off ramp”).
  - If “yes,” at what concentrations does that bioactivity occur? Whether or not those concentrations are likely to occur in humans is an element of risk assessment and not directly addressed here.
- If this substance is bioavailable and bioactive on biological targets that mediate cardiac contractile function, does that bioactivity result in adverse consequences for the host?
  - If not, the answer to the primary question is “no” and further assessment of cardiac endpoints is not necessary (an “off ramp”).
  - If the answer is “yes,” at what concentrations does that adverse bioactivity occur?
- If this xenobiotic is bioavailable, bioactive, and results in adverse consequences to the host, which hosts are most likely to be affected?

The articulation of specific questions, such as those detailed above, are typically not clearly stated a priori, and mapping of the associated biology for target organs of primary toxicological concern is not always the central focus in assay development or selection. Table S-2 shows the potential application of the pillars for evaluating potential cardiovascular toxicity by candidate pharmaceuticals.

Current in vitro systems do not fully represent the complexity of human biology for any particular target organ system. However, an important consideration is: *What level of completeness is required to make decisions throughout the decision-making continuum?* It is possible to identify salient biological features of specific organ systems that can enable the collection of informative data in decision-making contexts in much more scalable in vitro systems. Regarding cardiotoxicity, the question is: *How much do I need to know about cardiac bioactivity to inform the design of a cardiac-safe pesticide or drug, prioritize chemicals under TSCA, select a lead molecule with the best opportunity for an approvable risk:benefit profile or even receive Investigational New Drug (IND) approval.*

Table S-2. Example BQBA Implementation Scenario: Contractile Cardiotoxicity by a Pharmaceutical

| **Pillar** | **Key/Primary Question** | **Example Secondary Questions** | **Example Assessment/Tests/Strategies** |
| --- | --- | --- | --- |
| **Bioavailability** | Does the substance access the biological system? | - - - - Does the substance enter circulation?       - Is the substance metabolized to any intermediates in the body?       - Can the substance enter target cells/tissues (i.e., heart)? | - - - - Physicochemical properties (e.g., molecular weight, solubility, pKa, logP)       - ADME |
| **Bioactivity** | Does the substance have a biological effect on cellular components of the heart? | - - - - Does the substance directly or indirectly alter biological targets that mediate cardiac contractile function? | Assays for cardiac contractility-relevant targets   - - - - Β_1_ adrenergic receptor       - Adenosine receptor       - Endothelin receptor       - L-type Ca^2+^ channel       - Mitochondrial ATP production |
| **Adversity** | Does the substance have the potential to cause harm? | - - - - Does the bioactivity result in adverse effects in the host? | Cardiac contractility-relevant endpoints tested in vitro   - - - - Cardiomyocyte cytolethality       - Ca^2+^ transients       - Cardiomyocyte contraction   Cardiac contractility-relevant endpoints tested in vivo   - - - - Repeat-dose animal study evaluating mass and morphology       - Single-dose animal functional study of left ventricular pressure or QA interval |
| **Susceptibility** | Is there differential sensitivity to potential harm within a population? | - - - - Is there an increased risk for adversity relative to the general population that is due to intrinsic factors?       - Which hosts are most likely to be affected? | - - - - Developmental and reproductive studies       - Sex as a biological variable       - Animal models with pre-existing cardiometabolic conditions |

## Exemplar 2: Application of a BQBA to evaluate potential endocrine disruption by a pesticide

All pesticides undergo rigorous safety testing according to guidelines varying by country and regulation. The potential for perturbation of the endocrine system in nontarget organisms, including humans, is a concern because of the modes of action for pesticidal effect of these chemicals (Stoker and Kavlock 2010). Thus, regulations exist to ensure that pesticides with demonstrated endocrine disrupting effects are not released into the environment. For example, the EPA Endocrine Disruptor Screening Program (EDSP) is mandated to use validated methods to test chemicals for potential endocrine disruption (EPA 2009; 2011) and incorporates an alternative scientific approach to screen chemicals for endocrine system perturbation (EPA 2015). Below, the assays from the two-tiered testing approach of the EDSP is mapped to a BQBA to demonstrate how an existing approach fits (or does not fit) with the approach presented herein. Table S-3 shows the potential application of the pillars for testing for endocrine disruption by a pesticide.

Table S-3. Example BQBA Implementation Scenario: Endocrine Disruption by a Pesticide

| **Pillar** | **Key/Primary Question** | **Example Secondary Questions** | **Example Assessment/Tests/Strategies** |
| --- | --- | --- | --- |
| **Bioavailability** | Does the substance access the biological system? | - - - - Is this substance bio-persistent or bio-accumulative?       - Does it produce a persistent metabolite?       - Does the substance enter circulation?       - Can the substance enter target cells/tissues (e.g., thyroid, testes)? | - - - - Physicochemical properties (e.g., molecular weight, solubility, pKa, logP)*       - ADME*   **Not a part of the EDSP approach* |
| **Bioactivity** | Does the substance induce a host response? | - - - - Does the substance interact with the endocrine system (e.g., estrogen, androgen, thyroid hormone systems)? | US EPA EDSP Tier 1 screening assays:  **In vitro**   - - - - Estrogen receptor (ER) binding (rat uterine cytosol)       - Estrogen receptor (hERα) transcriptional activation (human cell line [HeLa-9903])       - Androgen receptor (AR) binding (rat prostate cytosol)       - Steroidogenesis (human cell line [H295R])       - Aromatase inhibition (human recombinant microsomes)   **In vivo**   - - - - Uterotrophic (rat)       - Hershberger (rat)       - Pubertal female (rat)       - Pubertal male (rat)       - Amphibian metamorphosis (frog)       - Fish short-term reproduction   **In silico**   - - - - ToxCast models for bioactivity (Tier 1 battery alternative) |
| **Adversity** | Does the substance have the potential to cause harm? | - - - - Are there adverse endocrine-related effects caused by exposure to the substance? | - - - - Review and integrate results from Tier 1 to determine adversity based on bioactivity   US EPA EDSP Tier 2 tests:   - - - - Avian two-generation toxicity test (Japanese quail)       - Medaka extended one-generation reproduction test       - Larval amphibian growth and development assay |
| **Susceptibility** | Is there differential sensitivity to potential harm within a population? | - - - - Is there an increased risk for adversity relative to the general population that is due to intrinsic factors such as         - particular life stages (i.e., puberty)?         - certain genotypes/genetic polymorphisms? | - - - - Tier 1 and 2 tests that address sensitive life stages:         - Pubertal female (rat)         - Pubertal male (rat)         - Amphibian metamorphosis (frog)         - Avian two-generation toxicity test (Japanese quail)         - Medaka extended one-generation reproduction test         - Larval amphibian growth and development assay         - Fish short-term reproduction       - Results of Tier 2 testing will be integrated with other hazard information and exposure assessment of the substance; risk assessment will include a dose-response analysis and application of appropriate safety factors for susceptibility |

## Exemplar 3: Application of a BQBA to evaluate potential neurodevelopmental toxicity of a substance

Although traditional animal testing guidelines exist for developmental neurotoxicity (DNT) studies, such studies are impractical or impossible for large chemical classes due to resource and time constraints. To address such a limitation, rapid biologically relevant screening approaches are needed. Such approaches result in prioritization of potential neurotoxic substances for further testing conducted in animals, if necessary, while deprioritizing others. A single assay is unlikely to address the complexity of neurodevelopment and the various distinct cellular processes that are involved (Behl et al. 2019). Further, the underlying cellular processes and molecular mechanisms of DNT or neurobehavioral effects are not as well understood as other toxicological endpoints or outcomes, contributing to the challenge of developing assays to effectively measure the likelihood of a substance to present a neurodevelopmental or neurobehavioral hazard. It has been shown that several NAMs (e.g., zebrafish) may be more predictive of neurodevelopmental effects in humans than rodents (Behl et al. 2019). Table S-4 shows the potential application of the pillars for neurodevelopmental hazard characterization, albeit an area of assay development and data validation.

Table S-4. Example BQBA Implementation Scenario: Neurodevelopmental Toxicity of a Substance

| **Pillar** | **Key/Primary Question** | **Example Secondary Questions** | **Example Assessment/Tests/Strategies** |
| --- | --- | --- | --- |
| **Bioavailability** | Does the substance access the biological system? | - - - - Is this substance bio-persistent or bio-accumulative?       - Does the substance enter circulation?       - Can the substance cross the blood-brain barrier? | - - - - Physicochemical properties (e.g., molecular weight, solubility, pKa, logP)       - ADME/model predictions: blood-brain barrier permeability |
| **Bioactivity** | Does the substance induce a host response? | - - - - Does the substance interact with the brain? | - - - - Neurotransmitter receptor binding or activation assays (cell free)       - Alterations in expression of genes related to neurodevelopmental or behavioral processes       - Damage to brain cells (e.g., oxidative stress) |
| **Adversity** | Does the substance have the potential to cause harm? | - - - - Are there adverse effects on neurodevelopment caused by exposure to the substance? | *Test battery under development*   - - - - Neurite outgrowth: human iPSCs       - Neuronal network formation: rat primary cortical cells       - Behavioral assay models: zebrafish and planaria |
| **Susceptibility** | Is there differential sensitivity to potential harm within a population? | - - - - Is there an increased risk for adversity relative to the general population that is due to intrinsic factors such as         - particular life stages (i.e., specific stages of development)?         - certain genotypes/genetic polymorphisms? | - - - - Animal models specific to developmental life stages of humans |
